# Supplementary material for: Examining Indigenous Identity as a Protective Factor in Mental Well-Being Research in the United States: A Scoping Review
Source: Int J Environ Res Public Health. 2024 Oct 24;21(11):1404. doi: 10.3390/ijerph21111404 (PMC11594160; doi:10.3390/ijerph21111404)
Supplement: Supplementary file 1 [file ijerph-21-01404-s001.zip › ijerph-3203180-supplementary.pdf]

Concept Search Table and Data Extraction Tool

Concept Search Table

Database: PubMed

	Concept 1: Identity	Concept 2: Mental Health + Stress	Concept 3: Population of Interest
Key words	identity<br>OR<br>cultur*<br>OR<br>ethnic*<br>OR<br>trib*<br>OR<br>"blood quantum"<br>OR<br>racism	mental health<br>OR<br>anxiety<br>OR<br>“anxiety disorder”<br>OR<br>“generalized anxiety disorder”<br>OR<br>stress<br>OR<br>"acute stress"<br>OR<br>"episodic stress"<br>OR<br>"chronic stress"<br>OR<br>"perceived stress"<br>OR<br>"acculturative stress"<br>OR<br>"occupational stress"<br>OR<br>“mental exhaustion”<br>OR<br>“emotional exhaustion”	Indigenous<br>OR<br>“Native Hawaiian”<br>OR<br>“Native American”<br>OR<br>“American Indian”<br>OR<br>“Alaska Native”
Results (Keywords, not MeSH):	**2,407,681**	**1,890,344**	**66,743**
Results from all categories:	**3,173**		

(identity OR cultur* OR ethnic* OR trib* OR "blood quantum" OR racism)

AND

(mental health OR anxiety OR “anxiety disorder” OR “generalized anxiety disorder” OR stress OR "acute stress" OR "episodic stress" OR "chronic stress" OR "perceived stress" OR "acculturative stress" OR "occupational stress" OR “mental exhaustion” OR “emotional exhaustion”)

AND

(Indigenous OR “Native Hawaiian” OR “Native American” OR “American Indian” OR “Alaska Native”)

= 3,173

CINAHL:

	Concept 1: Identity	Concept 2: Mental Health + Stress	Concept 3: Population of Interest
Key words	identity<br>OR<br>cultur*<br>OR<br>ethnic*<br>OR<br>trib*<br>OR<br>"blood quantum"<br>OR<br>racism	mental health<br>OR<br>anxiety<br>OR<br>“anxiety disorder”<br>OR<br>“generalized anxiety disorder”<br>OR<br>stress<br>OR<br>"acute stress"<br>OR<br>"episodic stress"<br>OR<br>"chronic stress"<br>OR<br>"perceived stress"<br>OR<br>"acculturative stress"<br>OR<br>"occupational stress"<br>OR<br>“mental exhaustion”<br>OR<br>“emotional exhaustion”	Indigenous<br>OR<br>“Native Hawaiian”<br>OR<br>“Native American”<br>OR<br>“American Indian”<br>OR<br>“Alaska Native”
Results (Keywords, not MeSH):	**391,848**	**509,773**	**24,430**
Results from all categories in academic journals:	**1,306**		

All sources on CINAHL

(identity OR cultur* OR ethnic* OR trib* OR "blood quantum" OR racism)

AND

(mental health OR anxiety OR “anxiety disorder” OR “generalized anxiety disorder” OR stress OR "acute stress" OR "episodic stress" OR "chronic stress" OR "perceived stress" OR "acculturative stress" OR "occupational stress" OR “mental exhaustion” OR “emotional exhaustion”)

AND

(Indigenous OR “Native Hawaiian” OR “Native American” OR “American Indian” OR “Alaska Native”)

= 1,393

PsyInfo:

	Concept 1: Identity	Concept 2: Mental Health + Stress	Concept 3: Population of Interest
Key words	identity<br>OR<br>cultur*<br>OR<br>ethnic*<br>OR<br>trib*<br>OR<br>"blood quantum"<br>OR<br>racism	mental health<br>OR<br>anxiety<br>OR<br>“anxiety disorder”<br>OR<br>“generalized anxiety disorder”<br>OR<br>stress<br>OR<br>"acute stress"<br>OR<br>"episodic stress"<br>OR<br>"chronic stress"<br>OR<br>"perceived stress"<br>OR<br>"acculturative stress"<br>OR<br>"occupational stress"<br>OR<br>“mental exhaustion”<br>OR<br>“emotional exhaustion”	Indigenous<br>OR<br>“Native Hawaiian”<br>OR<br>“Native American”<br>OR<br>“American Indian”<br>OR<br>“Alaska Native”
Results (Keywords, not MeSH):	**679,443**	**1,174,649**	**28,311**
Results from all categories in academic journals:	**3,758**		

All sources in PsychInfo

(identity OR cultur* OR ethnic* OR trib* OR "blood quantum" OR racism)

AND

(mental health OR anxiety OR “anxiety disorder” OR “generalized anxiety disorder” OR stress OR "acute stress" OR "episodic stress" OR "chronic stress" OR "perceived stress" OR "acculturative stress" OR "occupational stress" OR “mental exhaustion” OR “emotional exhaustion”)

AND

(Indigenous OR “Native Hawaiian” OR “Native American” OR “American Indian” OR “Alaska Native”)

= 5,422

Data Extraction Tool

**Scoping Review Details**	
Scoping Review title:	Tools for Measuring the Impact of Indigenous Peoples within the United States' Ethnic Identities on Psychosocial Stressors
Review objective/s:	• Aim1: To determine where researchers are conducting their research in the United States: in which regions, and with which specific Indigenous populations<br>• Aim 2: To determine the number of tools being utilized when conducting research with Indigenous populations and measuring ethnic identity and describe these tools<br>o Aim 2.1: Determine the variables included in each data collection tool<br>• Aim 3: To better understand what is known about the relationship between Indigenous ethnic identity and mental wellbeing
Review question/s:	What tools currently exist to measure Indigenous ethnic identity for United States Indigenous populations in studies on psychosocial stressors?
**Author Information**	
Author names	
Year of publication	
Objective of paper	
**Inclusion/Exclusion Criteria**	
Population	
Concept	

Context	
Types of evidence source	
**Evidence source**<br><br>**Details and Characteristics**	
Citation details (e.g. author/s, date, title, journal, volume, issue, pages)	
Study Design	
Research question	
Tribe/s involved	
Urban area/s involved	
Participants: total number	
Participants: Age	
Participants: Gender	
Outcomes reported	
Identity Scale/s used	
Stress Scale/s used	
Results	
Conclusion of study	
Other relevant details	
**Details/Results extracted from source of evidence** (in relation to the concept of the scoping review)	
What is the name of the tool?	
E.g. Number of items in tool	
